# Innervation is higher above Bone Remodeling Surfaces and in Cortical Pores in Human Bone: Lessons from patients with primary hyperparathyroidism

**DOI:** 10.1038/s41598-019-41779-w

**Published:** 2019-03-29

**Authors:** Manasi Sayilekshmy, Rie Bager Hansen, Jean-Marie Delaissé, Lars Rolighed, Thomas Levin Andersen, Anne-Marie Heegaard

**Affiliations:** 1 Department of Drug Design and Pharmacology, University of Copenhagen, Copenhagen, Denmark; 2 Department of Clinical Cell Biology, Vejle Hospital – Lillebaelt Hospital, Institute of Regional Health Research, University of Southern Denmark, Vejle, Denmark; 3 Department of Surgery and Department of Otorhinolaryngology, Aarhus University Hospital, Aarhus, Denmark; 4 Clinical Cell Biology, Research Unit of Pathology, Department of Clinical Research, University of Southern Denmark, Odense University Hospital, Odense, Denmark; 5 Department of Forensic Medicine, Aarhus University, Aarhus, Denmark

## Abstract

Mounting evidence from animal studies suggests a role of the nervous system in bone physiology. However, little is known about the nerve fiber localization to human bone compartments and bone surface events. This study reveals the density and distribution of nerves in human bone and the association of nerve profiles to bone remodeling events and vascular structures in iliac crest biopsies isolated from patients diagnosed with primary hyperparathyroidism (PHPT). Bone sections were sequentially double-immunostained for tyrosine hydroxylase (TH), a marker for sympathetic nerves, followed by protein gene product 9.5 (PGP9.5), a pan-neuronal marker, or double-immunostained for either PGP9.5 or TH in combination with CD34, an endothelial marker. In the bone marrow, the nerve profile density was significantly higher above remodeling surfaces as compared to quiescent bone surfaces. Ninety-five percentages of all nerve profiles were associated with vascular structures with the highest association to capillaries and arterioles. Moreover, vasculature with innervation was denser above bone remodeling surfaces. Finally, the nerve profiles density was 5-fold higher in the intracortical pores compared to bone marrow and periosteum. In conclusion, the study shows an anatomical link between innervation and bone remodeling in human bone.

## Introduction

Bone is increasingly recognized as an organ in dynamic interaction with the rest of the body. Accordingly, local bone events such as bone resorption and formation seem to be tightly controlled through endocrine regulatory loops and the nervous system^1,2^. Nearly two decades ago animal studies showed that, in addition to autocrine and paracrine signaling, there was a leptin-dependent central control of the osteoclasts and osteoblasts^3,4^. Importantly, this hypothalamic regulation was mediated through β2 adrenergic signaling by the sympathetic nervous system^4,5^. Even before identifying this central control on the bone, earlier innervation studies suggested a neuronal regulatory system controlling skeletal development and bone cell functions. In rats, both sensory and autonomic innervations were demonstrated during heterotrophic bone formation, indicating that new bone formation is influenced by neuronal signaling^6^. Experimental denervation of the saphenous and sciatic nerves reduced skeletal growth of the metatarsal bones in rat pups^7^; and in neonatal rats, guanethidine-induced sympathetic denervation increased the mandibular periosteal surface occupied by osteoclasts, while capsaicin-induced sensory denervation decreased the surface occupied by osteoclasts in response to maxillary tooth extraction^8^. Meanwhile, in adult rats, an increase in the numbers of osteoclasts was showed after capsaicin-induced sensory denervation at the dorsal spine level leading to increased bone resorption^9^. These denervation studies confirmed the role of innervation in skeletal physiology. In addition, nerve fibers in the bone marrow of animals are predominantly associated with vasculature^10–13^; and vasculature has been shown to be important for skeletal growth, development and repair^14,15^. In fact, the association of nerves and vasculature are well-known and have been shown to have a similar wiring mechanism, which supports each other^16^. Therefore, it is likely that the neuronal and vascular networks have a similar functional association in the human bone. Innervation of the bone marrow by sympathetic nerves is also important in the migration of the hematopoietic stem cells and progenitor cells (HSPC) from the bone marrow niche as shown in mice deficient in UDP-galactose ceramide galactosyltransferase owing to poor conduction velocities of the nerves thus leading to less mobilization of the hematopoietic stem cells and progenitor cells^17^. Neuropeptides are shown to have a direct effect on the proliferation and activity of osteoclast and osteoblasts. Vasoactive intestinal peptide (VIP) was found important for the mobility and bone resorbing abilities of osteoclast^18^, and substance P (SP) and calcitonin gene-related peptide (CGRP) stimulated bone formation rates and osteoblast proliferation^19,20^. In addition, co-cultures of osteoclasts and osteoblasts in CGRP-containing media decreased the numbers of resorption lacunae, suggesting an inhibitory effect of CGRP on osteoclasts^21^. The absence of SP in tachykinin 1 deficient mice was shown to decrease the number of mature osteoclasts, but to a greater degree impair the survival of osteoblasts compared to wild type mice resulting in a net bone loss^22^. A delay in callus differentiation, reduction in bone mechanical properties and alterations in bone microarchitecture was also shown in mice deficient of SP and sympathetic nervous system^23^. SP and Neuropeptide Y have also been shown to control the integrity of blood vessels and the mobility of HSPC in the bone marrow^24^. Whether neuropeptides released by nerves fibers within the bone marrow are in proximity to affect the osteoclasts and osteoblasts involved in the human bone remodeling remains an open question.

Clinical observations from patients with spinal cord injury, head injury and stroke have demonstrated changes in fracture healing, increased fragility of bone and increased callus formation^25–27^. Although this may be caused by a decrease in the mechanical stimulation, due to mobility impairment, it may also in part be caused by alterations in the nervous system impairing human bone metabolism. Also, case studies of children suffering from mutations in the gene coding for the nerve growth factor receptor (TrkA) report short stature, teeth loss, delayed fracture healing, as well as insensitivity to pain^28–30^. Human bone is highly adaptive to mechanical load, exemplified by the reduction of bone mass in astronauts under microgravity^31^. This bone loss may likely be neuronally regulated, as shown to be the case in functional adaptation studies to loading and unloading in rats and mice^32–34^.

Thus we hypothesize that bone remodeling surfaces in human bones should present an increased density of innervation in the neighboring bone marrow compared to quiescent surfaces. An anatomical association of nerves to the sites of bone remodeling has never been shown before. To increase the chances of detecting remodeling sites, this study was performed on iliac crest biopsies isolated from patients with primary hyperparathyroidism (PHPT), a condition where bone remodeling is increased with more than 50%^35^. In the investigated human biopsies, the eroded and formation surfaces are believed to reflect bone remodeling events, where bone resorption is followed by bone formation in a highly orchestrated process^34,36^. Furthermore, vascularization is also known to be important in regulating skeletal growth and bone remodeling, and thus we look into the neuronal association of the vascular structures in the bone marrow and close to the bone remodeling surfaces.

Finally, very little is known about the distribution of nerve fibers to local compartments in human bone. Therefore the present study also investigates the distribution of nerve fibers in human bone. It describes the density of nerve profiles in the periosteum, cortical pores and bone marrow of human iliac crest biopsies, discriminating between nerve profiles with or without sympathetic nerve fibers.

## Results

### The differential density of innervation in human bone sub-compartments

**Figure 1:**
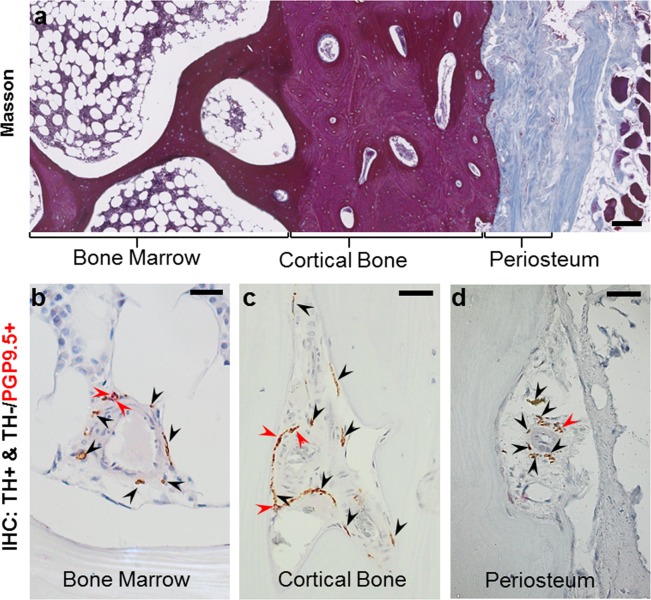
Illustration of the method used for the analysis of nerve profiles in human bone. (**a**) Masson’s trichrome stained section showing the overall compartmentalization of the iliac crest biopsy into bone marrow, cortical bone, and periosteum. Sections were sequentially immunostained for tyrosine hydroxylase (TH, black arrows) and protein gene product 9.5 (PGP9.5, red arrows). The images illustrate this immunostaining within the bone marrow (**b**), cortical pore (**c**) and periosteum (**d**). The sequential immunostaining allows discrimination between the TH+ nerve profiles with sympathetic nerve fibers (brown) and the TH−/PGP9.5+ nerve profiles without sympathetic nerve fibers (red). Scale bars are 200 µm (**a**) and 50 µm (**b–d**).

To study differences in innervation among various bone compartments and to identify the association of the nerves to vasculature, the nerve profile density per area was estimated in the bone marrow, cortical pores and the periosteum of the iliac crest biopsies from PHPT patients (Fig. 1a). Thin sections were sequentially stained for tyrosine hydroxylase (TH) and the pan-neuronal marker protein gene product 9.5 (PGP9.5) to identify the sympathetic (TH+) and non-sympathetic (TH−/PGP9.5+) nerve profiles, respectively (Fig. 1b–d). Histomorphometric analysis was conducted on scanned images of the sections; identified nerve profiles were marked up and the areas of the three bone compartments were estimated using a point grid. Please refer to the method section for detailed descriptions of the methods used. The patients’ age, sex, areal bone mineral density (aBMD) T-scores and parathyroid hormone (PTH) levels obtained from the patient journal are reported in Table 1.

**Table 1:** Patient data.

Patient	Gender	Age	T-Score	T-Score	PTH
		(Years)	Lumbar Spine	Femur Neck	(pmol/l)
1	Female	64	−3,0	−0,8	12,2
2	Female	78	−0,7	0,2	35,0
3	Female	59	−0,5	−0,2	20,0
4	Female	60	−2,6	−2,2	33,5
5	Male	69	−0,4	0,0	17,4
6	Female	52	−2,3	−0,9	16,3
7	Female	58	−1,4	−0,6	13,3
8	Female	58	−1,8	−2,2	24,8
9	Female	60	−2,7	−1,1	25,6
10	Female	45	−0,9	−0,7	19,0
11	Female	57	−1,1	−0,8	18,9

*T-score: Normal > −1.0; Osteopenia < −1.0, >−2.5; Osteoporosis < −2.5.

*PTH: Normal range 1.6–6.9.

**Figure 2:**
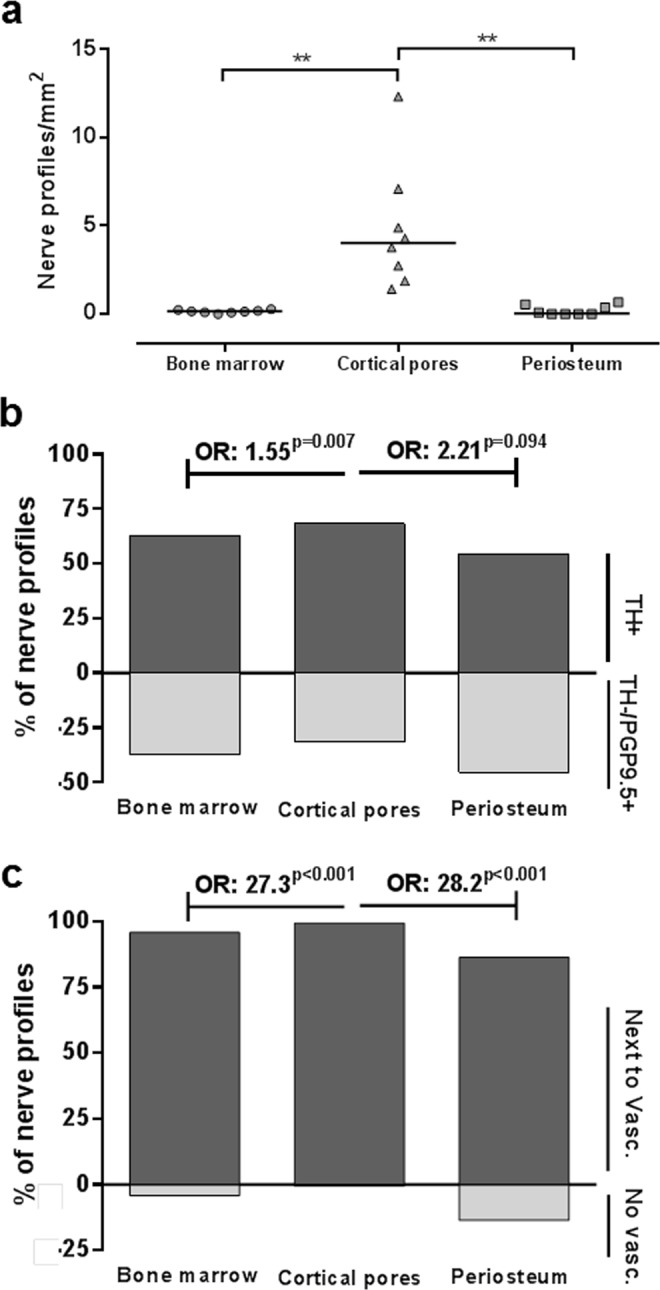
Nerve profile density in bone sub-compartments and their association to the vasculature. (**a**) The number of nerve profiles per tissue area was significantly higher in the cortical pores compared to the bone marrow and periosteum. Each dot represents the value in a given biopsy (n = 10), and the horizontal line represents the mean. The data were analyzed by repeated measure ANOVA with Geisser-Greenhouse’s correction: **p < 0.01. (**b**) The percentage of TH+ nerve profiles relative to TH−/PGP9.5+ nerve profiles was higher in the cortical pores compared to the bone marrow and periosteum (not significant). The data was analyzed by a clustered logistic regression, addressing whether the odds between TH+ and TH−/PGP9.5+ nerve profiles were different between the compartments. (**c**) Most of the nerve profiles were closely associated with the vasculature. Still, the percentage of nerve profiles associated with vasculature relative to those without a vascular association was higher in the cortical pores compared to the bone marrow and periosteum. The data was analyzed by a clustered logistic regression, addressing whether the odds between nerve profiles with or without vascular association is different between the compartments. TH: tyrosine hydroxylase, PGP9.5: protein gene product 9.5, OR: odds ratio.

Overall, the cortical pores were found to be significantly more densely innervated compared to both the periosteum and the bone marrow, demonstrating a 5-fold higher nerve profile density (Fig. 2a). This pattern of a higher nerve profile density in the cortical pores compared to both the periosteum and the bone marrow was consistently observed in all patients. No difference was observed in the density of innervation between the bone marrow and the periosteum.

Furthermore, the nerve profiles in the cortical pores were more often TH+ than TH−/PGP9.5+ compared to the nerve profiles in the bone marrow and periosteum (Fig. 2b). This suggests that a larger fraction of the nerve profiles contain sympathetic fibers in the cortical pores, than in the bone marrow and periosteum.

### Nerve profiles were associated with vasculature

The majority of the nerve profiles were morphologically observed to be associated with vasculature structures in all bone sub-compartments in the combined TH and PGP9.5 immunostaining (Fig. 2c). Still, the association with vascular structures was significantly higher in the cortical pores compared to the periosteum and bone marrow (Fig. 2c).

**Figure 3:**
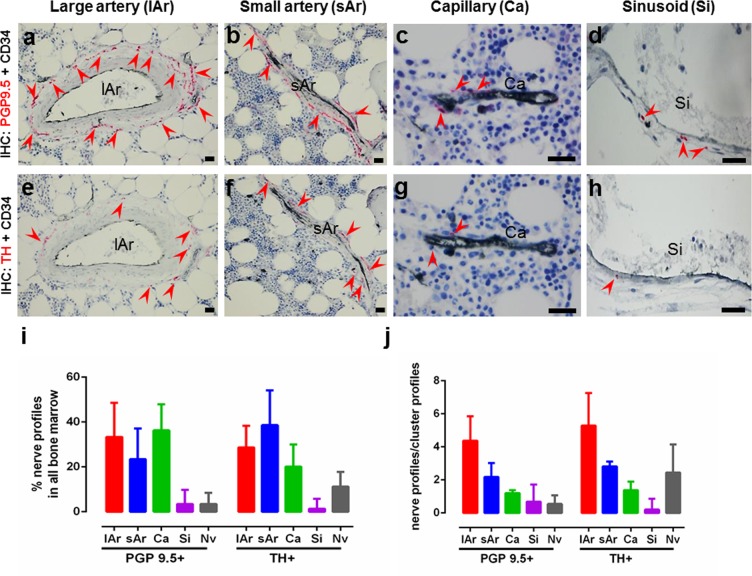
Association of nerve profiles to the vasculature. (**a–h**) Images showing double immunostaining of CD34+ vasculature in combination with PGP9.5+ or TH+ nerve profiles (red arrows) in the bone marrow. Large arteries with PGP9.5+ (**a**) or TH+ (**e**) nerve profiles, small arteries with PGP9.5+ (**b**) or TH+ (**f**) nerve profiles, capillaries with PGP9.5+ (**c**) or TH+ (**g**) nerve profiles, and sinusoids with PGP9.5+ (**d**) or TH+ (**h**) nerve profiles. (**i**) The percentage distribution of PGP9.5+ and TH+ nerve profiles associated with the respective vascular structures in the bone marrow, and (**j**) the number of nerve profiles/cluster profile for the respective vascular structures. TH: tyrosine hydroxylase, PGP9.5: protein gene product 9.5; lAr: large arteriole; sAr: small arteriole; Ca: capillary; Nv: Nerve profiles with no association to the vasculature. n = 11.

To further investigate the association of nerve profiles to given vascular structures in the bone marrow, sections were double-immunostained with either PGP9.5 or TH in combination with CD34, a vascular marker of endothelial cells, thus classifying the bone marrow blood vessels into capillaries, small and large arteries and sinusoids based on the size, morphology and staining of the respective vascular structures (Fig. 3a–h). Overall, the TH+ and PGP9.5+ nerve profiles were found to be mostly associated with large arteries, small arteries or capillaries, and to less extent associated with sinusoids (Fig. 3i). Only a few PGP9.5+ and TH+ nerve profiles were not associated with vascular structures (0.5% and 10%), respectively. However, it should be noted that nerve profiles can be 3-dimensionally associated with vascular structures above or below the plane of the section. Each individual vascular structure surrounded by nerve profiles was defined as a cluster profile. As expected, the number of nerve profiles per cluster profile was highest for large arteries, lower for small arteries and even lower for capillaries (Fig. 3j).

### Nerve profiles are more abundant close to the bone surfaces

**Figure 4:**
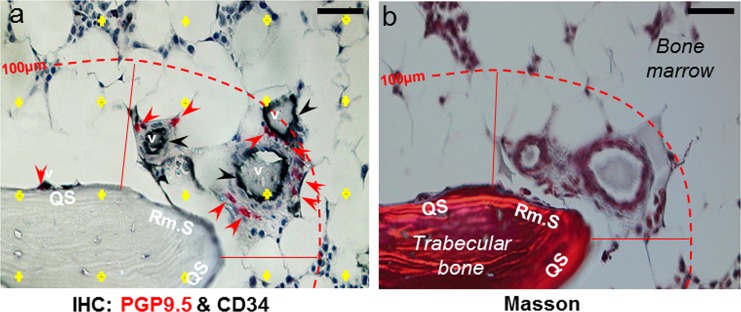
Illustration of the method used for the analysis of nerve profiles association to bone surfaces in human bone. **(a–b**) Adjacent sections were double-immunostained with either TH or PGP9.5 in combination with CD34, a marker of endothelial cells (**a**) or Masson’s trichrome stained (**b**). The images illustrate two adjacent sections. (**b**) In the Masson’s trichrome stained section, eroded and quiescent surfaces were collectively marked as remodeling surfaces (Rm.S), and these markings were transferred to the adjacent immunostained section showing CD34+ vascular structures (v, black) above the remodeling surface, which is associated with multiple PGP9.5+ (red) nerve profiles. **(a)** For the analysis of nerve density, the bone marrow was separated into different zones: the bone marrow below or above 100 µm (hatched line) of the bone surface, as well as the bone marrow above quiescent surfaces (QS) or remodeling surfaces (Rm.S) below 100 µm. A point-grid (yellow crosses) was used to estimate the area of the zones, in which the nerve density was estimated. Scale bars 50 µm.

**Figure 5:**
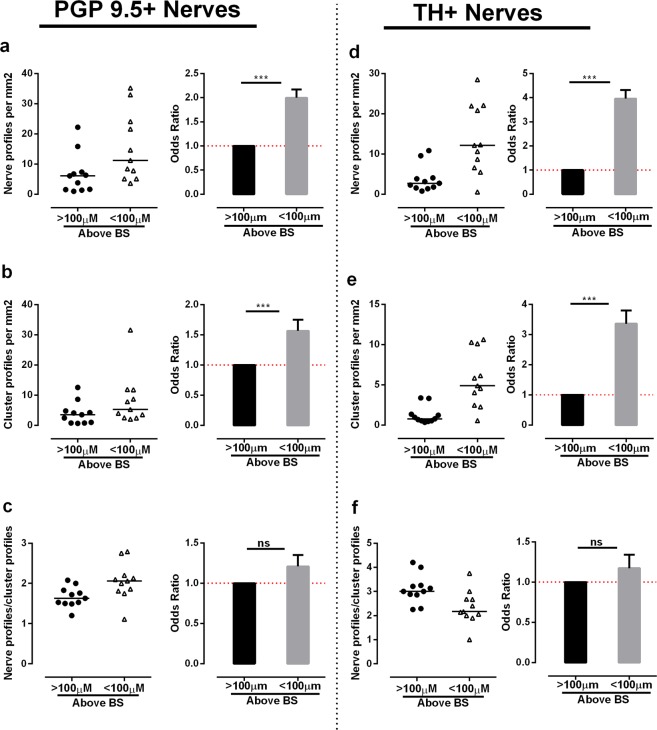
Nerve profiles are more abundant close to the bone surface. In the bone marrow, nerve and cluster profiles were quantified within and above 100 µm of the bone surfaces. **(a,d**) The density of PGP9.5+ (**a**) and TH+ (**d**) nerve profiles was significantly higher close to the bone surface. (**b,e**) The density of cluster profiles innervated by PGP9.5+ (**b**) and TH+ (**e**) nerve profiles was significantly higher close to the bone surface, indicating that innervated vascular structures are more abundant close to the bone surface compared to the distant bone marrow. (**c,f**) No difference was observed in the nerve profile/cluster profile ratio of the PGP9.5+ (**c**) and TH+ (**f**) innervation, indicating that the increase in innervation was not due to an increase in numbers of nerve profiles per vascular structure. The data was analyzed by clustered regression analysis. ***p < 0.001, n = 11. TH: tyrosine hydroxylase, PGP9.5: protein gene product 9.5.

In the bone marrow, the density of nerve profiles was not homogeneous. To address this heterogeneity, the density of all nerve profiles (PGP9.5+) and the nerve profiles with sympathetic nerve fibers (TH+) were estimated in the bone marrow within 100 µm of the bone surface and in the bone marrow >100 µm above bone surfaces (deep bone marrow), respectively (Fig. 4a). The number of PGP9.5+ and TH+ nerve profiles per marrow area was significantly higher within 100 µm of the bone surfaces compared to the number of nerve profiles per marrow area estimated in the deep bone marrow >100 µm above the bone surface (Fig. 5a,d). Since thin sections were used in the study, a nerve could branch out on a single plane and transect the plane multiple times and thus increase the chances of counting the branches as single nerve profiles. In order to eliminate the risk of obtaining a high nerve profile density by counting the same nerve profile multiple times, neuronal profiles were again investigated as cluster profiles. As the majority of neuronal profiles were associated with vasculature, it was decided to identify the cluster profiles as isolated vasculature structures with innervation. Similar to the individual nerve profiles, the number of TH+ and PGP9.5+ cluster profiles per marrow area was significantly higher close to the bone surface (within 100 µm) compared to the deep bone marrow (Fig. 5b,e). This shows that the density of innervated vasculatures was higher close to the bone surfaces compared to the density in the deep bone marrow. Note though, that this estimation does not allow any conclusions on the overall distribution of vasculature as only vasculature presenting with neuronal profiles were investigated. No difference was found between the number of nerve profiles/cluster profile for either the PGP9.5+ or the TH+ innervated clusters (Fig. 5c,f), indicating that the difference in innervation is not due to an increased number of neuronal profiles associated to the individual vascular structures. Of note, none of the estimated nerve densities showed any correlation with the T-Score or PTH level of the PHPT patients reported in Table 1 (data not shown).

Together, the analyses demonstrate that the overall density of nerve profiles and the density of nerve profiles with sympathetic nerve fibers are highest close to bone surfaces.

### Nerves profiles are more abundant over remodeling bone surfaces

**Figure 6:**
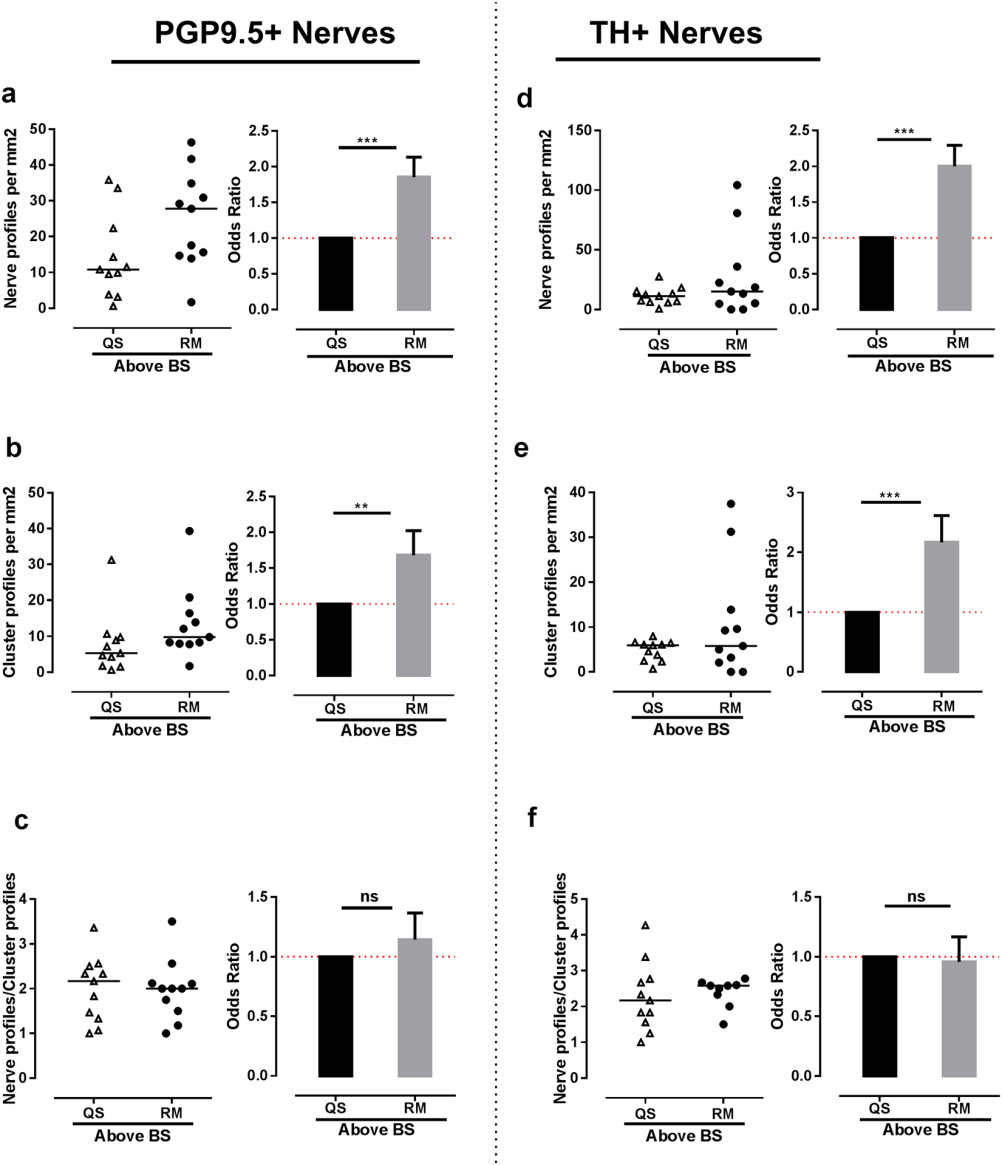
Nerve profiles are more abundant above bone remodeling surfaces. In the bone marrow, nerve and cluster profiles were quantified 0–100 µm above bone remodeling and quiescent surfaces. (**a,d**) The density of PGP9.5+ (**a**) and TH+ (**d**) nerve profiles was significantly higher above the remodeling surfaces compared to quiescent surfaces. (**b,e**) The density of cluster profiles innervated by PGP9.5+ (**b**) and TH+ (**e**) nerve profiles was significantly higher above bone remodeling surfaces. (**c,f**) No difference was observed in the nerve profile/cluster profile ratio of the PGP9.5+ (**c**) and TH+ (**f**) innervation, indicating that the increase in innervation was not due to an increase in numbers of nerve profiles per vascular structure. The data was analyzed by clustered regression analysis. **p < 0.01, ***p < 0.001, n = 11. TH: tyrosine hydroxylase, PGP9.5: protein gene product 9.5.

To further investigate the possible link of innervation to bone remodeling in human bone, the density of all nerve profiles (PGP9.5+) and nerve profiles with sympathetic nerve fibers (TH+) over quiescent and remodeling bone surfaces was estimated within 100 µm of the bone surface. The quiescent, eroded and formation surfaces were identified on adjacent Masson’s trichrome stained sections (Fig. 4b). Areas with breaks in the lamellae, which appear as bites or depression on the bone surface, were identified as eroded surfaces, while un-mineralized regions with collagen fibers appearing in blue color were identified as osteoid surfaces, and hence taken as formation surfaces (Fig. 4b). The study did not have sufficient power to address whether the nerve profile density over eroded and formation (osteoid) surfaces was different (data not shown). Hence, the eroded and osteoid surfaces were pooled as remodeling surfaces in the analysis. The number of PGP9.5+ nerve or cluster profiles per marrow area was significantly higher above remodeling surfaces than above quiescent surfaces (Fig. 6a,b). The same was shown for TH+ nerve and cluster profiles (Fig. 6d,e). No significant difference was found in the numbers of nerve profile/cluster profile for either PGP9.5+ or TH+ innervation above quiescent and remodeling surfaces (Fig. 6c,f). Again, none of the estimated nerve densities showed any correlation with the T-Score or PTH level of the PHPT patients reported in Table 1 (data not shown).

## Discussion

**Figure 7:**
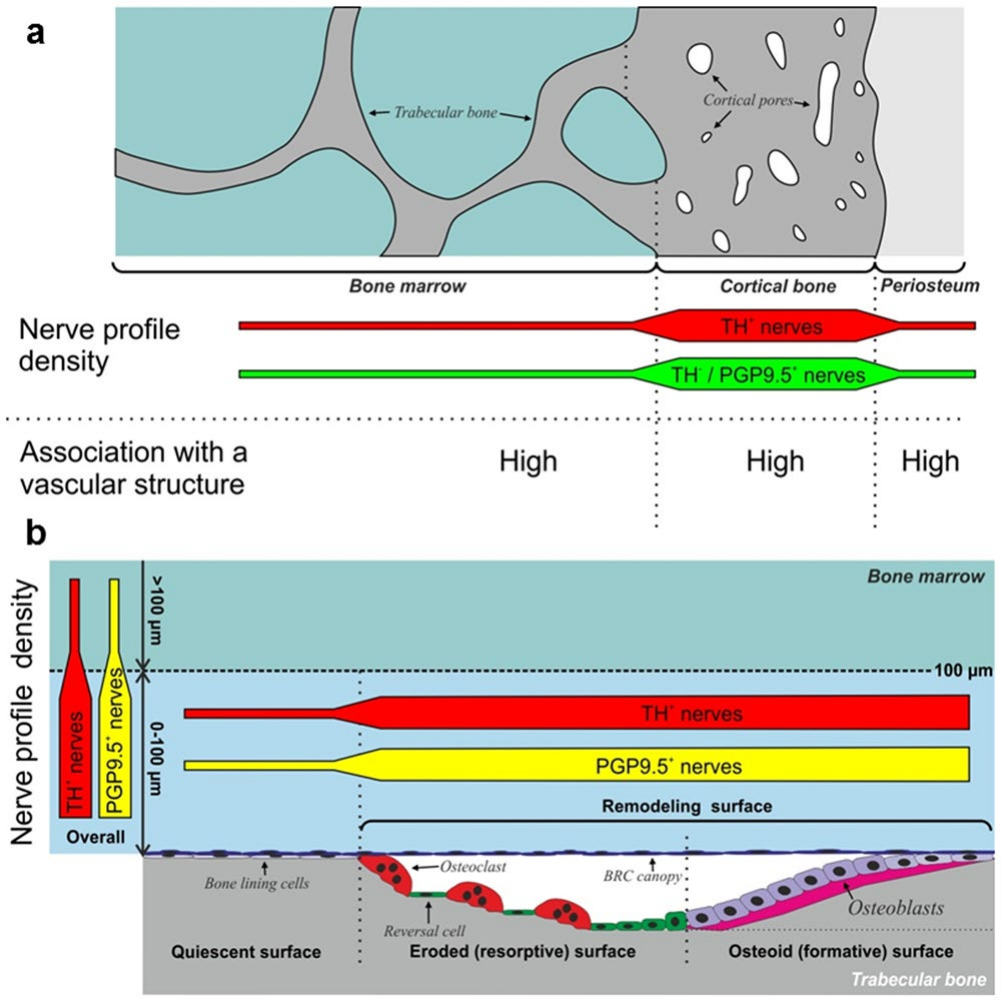
(**a**) Model illustrating the nerve profile density in the intra-cortical pores, periosteum and bone marrow. The nerve profile density was highest in the intracortical pores for both sympathetic (TH+) and non-sympathetic (TH−/PGP+) fibers. The majority of nerve profiles were associated with vascular structures. (**b**) Model illustrating the nerve profile density above bone quiescent and remodeling surfaces. In the bone marrow, the density of all nerve profiles (PGP9.5+) and sympathetic nerve profiles (TH+) was highest close to the bone surface (0–100 µm). The highest density of neuronal profiles was found above bone remodeling surfaces as compared to quiescent surfaces. TH: tyrosine hydroxylase, PGP9.5: protein gene product 9.5.

Limited data exist on the innervation of human bone, and the local association to bone resorption and formation sites of bone remodeling events. Therefore, we aimed to study the nerve distribution in human bone and its association to bone remodeling surfaces. The current study demonstrates that the local innervation is denser above the eroded and formation surfaces of remodeling events in the human bone marrow cavity, supporting the notion that nervous system plays a role in bone formation and resorption of human bone remodeling. Moreover, it shows that human bone is innervated by both sympathetic and non-sympathetic fibers that are strongly associated to the vascular network, and that there is a difference in the density of innervation within the innervated vascular networks and among the human bone compartments. The conclusions of the study are summarized in Fig. 7 and discussed in the paragraphs below.

We found that in human bone, the density of nerve profiles was significantly higher in the cortical pores compared to the periosteum and bone marrow, which had a similar density of nerve profiles. This is in contrast to the studies in rats and mice that report a significantly higher density of innervation in the periosteum compared to mineralized bone and bone marrow^37–39^. This discrepancy is not surprising since the intracortical pores in humans reflect an extended network of vascularized canals that are generated and modulated by intracortical bone remodeling events^40,41^. A network, which is much less evolved in rats and mice^42,43^. Also, the present human study investigates the innervation in the iliac crest, while the studies on mice and rats address the innervation in the femur^37–39^. We address nerve profile density in the cortical pores and not overall in the cortex, as we only observed nerves in the intracortical pores, not in the cortical bone matrix, supporting that it seems more relevant to divide the number of nerve profiles with the area of the intracortical pores. The observed magnitude and consistency of the increased nerve density within cortical pores versus bone marrow and periosteum strengthen the notion that cortical pores are the most densely innervated sub-compartment of the human bone, at least in the iliac crest. Future studies, using the procedures developed in this study, are warranted to address whether these densities are similar in bones of healthy individuals, may vary between different skeletal sites and sex, or may be affected under different pathophysiological conditions or treatments.

Animal studies demonstrated that all bone compartments are innervated by both sensory and sympathetic nerve fibers^11,37,39^. Interestingly, a small increase was observed in the density of nerve profiles that contained TH+ sympathetic nerve fibers in the cortical pores compared to the bone marrow and periosteum, suggesting that the ratio between sympathetic and non-sympathetic fibers vary between the three sub-compartments of the human iliac crest biopsies. This was in contrast to a recent study published reporting a decline in the TH+ sympathetic nerve fibers in the aging cortical bone compared to the adult cortical bone of the mouse femur^44^. However, due to the structural differences of the human and mouse cortical bones, it is challenging to compare the innervation of the cortical bones in humans to the cortical bones in rodents^37–39^. It should also be taken into account that the age group of the patient cohort used is not equivalent to the age group of the rodent models often used for innervation studies.

Animal studies addressing the role of the nervous system in bone remodeling are emerging, but still, the majority of innervation studies have focused on the types of nerves and their distribution in bone sub-compartments^10–12,37–39^, rather than linking their anatomical association to bone remodeling surfaces. Moreover, most of these studies have used growing rodents that may have more bone modeling than remodeling^45,46^. Therefore the current study aimed to associate innervation to eroded and formative surfaces in human bone, which preferentially reflect human bone remodeling sites.

In order to increase the probability of finding these bone remodeling sites, the study was conducted on iliac crest biopsies obtained from patients diagnosed with primary hyperparathyroidism (PHPT), which is known to have an increased extent of active remodeling surfaces compared to controls^47–50^. This increased extent of open active remodeling sites in PHPT patients explains, in part, their reduced T-score in the lumbar spine and femur neck by Dual-energy X-ray absorptiometry (DXA) (Table 1). Here, a T-score between −1.0 to −2.5 standard deviations below the young adult female reference mean is defined as osteopenic, while a T-score more than −2.5 standard deviations below the young adult female reference mean is defined as osteoporotic^51–53^. The increased extent of open active remodeling sites in PHPT patients is due to a high endogenous level of PTH, which was 2–5 folds higher in the included patients than the reference range for healthy adults. Even though this study would have been extremely difficult to conduct on bone biopsies from healthy controls with a lower extent of active remodeling sites, one may still question whether findings in PHPT patients are transferrable to healthy controls. On the other hand, none of the estimated nerve profile densities in the bone marrow showed any correlation with the T-scores or PTH levels of the PHPT patients, supporting the notion that the findings in PHPT patients are likely transferrable to healthy controls.

The density of nerve profiles was first estimated in the bone marrow close to or away from the bone surface, and then in the bone marrow close to quiescent or remodeling surfaces. This showed that both the PGP9.5+ and TH+ nerve profiles were denser in the bone marrow close to bone surfaces, with the highest density above bone remodeling sites thus confirming our hypothesis that the nerve density in human bone is higher close to the remodeling surfaces. This increased density of nerve profiles above remodeling sites supports the anatomical link between the innervation and the coordinated bone resorption and formation of human bone remodeling sites. Moreover, the proximity of nerves to remodeling sites provides a physical rationale for the reduction of bone pain by inhibitors of osteoclastic bone resorption^54^.

The iliac crest biopsies were isolated from patients with PHPT, a condition which in general is reported to increase bone remodeling in trabecular and cortical bone. However, in the iliac crest, the trabecular volume and structure are found to be preserved in patients with PHPT, while increased bone remodeling in the cortical bone leads to increased cortical porosity and cortical bone loss^35,48,55,56^. As we evaluated the association of nerve profiles to bone surfaces above trabecular bone surfaces only, we do not expect that PHPT has a significant influence on the data compared to the physiological bone remodeling in trabecular bone. However, further studies are required to confirm this.

Other factors influencing bone remodeling and potentially also the nerve densities are age and sex^57–59^. Bone mass tends to decrease with age as a result of an overall imbalance in the bone resorption and bone formation. In women, bone loss is accelerated following menopause^57^. The 10 of the 11 PHPT patients used in this study are women either at menopause or postmenopausal (Table 1). One may speculate that age-induced changes in the nerve and vascular densities above remodeling may contribute to the overall imbalance between bone resorption and formation leading to bone loss. Moreover, one may question whether the distribution and densities of nerves in the bone marrow are different between women and men. Future studies investigating the nerve distribution and densities in a wide age-range of women and men may answer this question.

The nerve fibers associated with bone remodeling sites may release neuropeptides, which have been shown to affect the function of osteoblasts and osteoclasts like: CGRP, which has been shown to affect the bone resorption by osteoclasts, osteoblast proliferation and bone formation rates^20,60^; Substance P, which have been shown to induce osteoclastogenesis *in vitro*^61,62^; and VIP, which has been shown to affect osteoclasts mobility and bone resorption capabilities *in vitro*^18,63^. Also, glutamate is a known potent neurotransmitter involved in maintaining bone metabolism by having a functional effect on the osteoclast activities^64^. In this study, we found that all bone compartments were innervated by both sympathetic and non-sympathetic neuronal profiles. The non-sympathetic profiles could possibly be sensory nerve fibers, as expected from animal studies but further studies using specific markers are required to fully establish whether this is the case^11,12,65^.

Bone is a highly vascularized tissue and includes therefore, a vast vascular network, stretching from periosteum through the cortex to the marrow cavity^16,66,67^. Three-dimensional studies of the vasculature in the mouse femur demonstrated that this vascular network starts as large arteries which branched into small arteries and capillaries, which finally transited into sinusoids^68^. A similar, but more elaborated vascular network is believed to exist in the human bone^69^. The present study demonstrates that the nerve profiles were preferentially associated with large and small arteries, as well as capillaries of the vascular network in the bone marrow. Of note, the association of nerve profiles to vascular structures was investigated from the innervation perspective only, and thus not based on the density or size of the specific vasculatures; i.e. the data does not provide any information on the total coverage of vascular structures.

Since the vascularization and innervation seem to be entwined, and both have been ascribed an important role in the regulation of bone resorption and formation^2,14,15^, one may argue that these two systems function together within the bone. In relation to bone remodeling, it is especially interesting that the present study shows that the density of cluster profiles, reflecting the density of innervated vascular structures, were significantly higher close to the bone remodeling surfaces. This is in line with a prior study demonstrating that the vascular structures, preferentially capillaries, are more abundant close to bone remodeling sites in human bone^69^. Here the vascular-associated nerve fibers may: (i) regulate the local blood flow to the remodeling sites, as reported to be the case in muscles and other organs^70^; (ii) direct the vasculatures to remodeling sites through the release of neuropeptides, e.g. Substance P, which has been reported to be involved in angiogenesis both *in-vitro* and *in-vivo*^71,72^; and (iii) regulate the vascular permeability, as well as the permeability of the bone marrow envelope, i.e. bone-remodeling compartment (BRC) canopy, above remodeling sites^73,74^. Here the permeability may be neuronally regulated in the same way as the regulation of blood-brain barrier permeability by CGRP and Substance P following neuronal inflammation in the central nervous system^75,76^.

Taken together, the close association of innervation and vasculature at the areas of bone remodeling, demonstrated in the current study, strongly suggests that vasculature and nerves in concert orchestrate the nourishments of local bone remodeling events. Future studies are warranted to further address the combined role of the vascular and neuronal network in local bone modeling and remodeling.

In conclusion, this study confirms that the innervation density is higher close to the areas of bone remodeling in human bones by showing a clear anatomical association of nerves to the bone remodeling surfaces. It also shows that the human iliac crest is innervated by both sympathetic and non-sympathetic nerves, with the highest density of nerve profiles found in the intracortical pores compared to the bone marrow and the periosteum. Moreover, the study shows that vasculatures with innervation are higher close to bone remodeling surfaces in the bone marrow. This finding is in agreement with the previously reported increased presence of capillaries next to bone remodeling sites in the bone marrow microenvironment^69^ and demonstrates a situation where the roles of vasculature and nerves may be entwined^16^. Collectively, the present study provides a micro-anatomical rationale for the neural regulation of the bone resorption and formation within human bone remodeling. Further studies are needed in order to clarify whether these findings reflect the innervation under physiological and other pathological conditions, as well as in other skeletal sites.

## Materials and Methods

### Human bone biopsies and sectioning

Seven-mm trans-iliac biopsies isolated from 11 patients with primary hyperparathyroidism (PHPT) were included in the study. The biopsies were obtained from patients during parathyroidectomy at the Department of Surgery and Department of Otorhinolaryngology, Aarhus University Hospital. The study was conducted on bone biopsies from PHPT patients due to their increased presence of bone surfaces with remodeling. The included PHPT patients were preferentially women (ten women and one man) with a mean age of 60 years (aged 45–78 years). The aBMD T-score by DXA and PTH values were obtained from the patient journal. Patient data are summarized in Table 1. The study was approved by the Danish National Committee on Biomedical Research Ethics (journal no. S-20070121) in compliance with the World Medical Association Declaration of Helsinki – Ethical Principle for Medical Research Involving Human Subjects. The study was conducted according to the rules and guidelines of the Danish National Committee on Biomedical Research Ethics (journal no. S-20070121) and informed consent was obtained from all the patients included in the study.

The biopsies were fixed in 4% paraformaldehyde for 24 hours at room temperature immediately after isolation, decalcified in 10% formic acid for 14–28 days and embedded in paraffin. Four sequential 3.5-µm-thick sections were cut from each biopsy. The first section (section 1) was Masson’s trichrome stained to discriminate between the quiescent and remodeling surfaces^73^, while the remaining three sections (section 2–4) were double-immunostained for neuronal and vascular markers.

### Double-immunostaining

The sections were deparaffinized, blocked for endogenous peroxidases, subjected to overnight antigen retrieval in Tris-EDTA buffer (pH 9.0) at 60 °C, and blocked with 1% casein in Tris-buffered saline to reduce unspecific binding.

Section 2 of each biopsy was first immunostained for tyrosine hydroxylase (TH) and subsequently for protein gene product 9.5 (PGP9.5) to identify the TH+ and TH−/PGP9.5+ nerve profiles. Although PGP9.5 is a known pan-neuronal marker and therefore should also be expressed by the TH+ nerve profiles thus making them TH+/PGP9.5+, we decided to classify these nerve profiles only as TH+. First, the sections were labeled with polyclonal rabbit anti-TH antibodies (AB158, Millipore), which were detected with polymeric horseradish peroxidase-conjugated anti-rabbit IgG (BrightVision DPVR-HRP, Immunologic), and visualized with diaminobenzidine (DAB+, Dako K3468). Second, the sections were labeled with polyclonal rabbit anti-PGP9.5 antibodies (SAB4503057, Sigma), which were detected with polymeric alkaline phosphatase-conjugated anti-rabbit IgG (BrightVision DPVR-AP, Immunologic), and visualized with Liquid Permanent Red (LPR; Dako K0640).

Section 3–4 of each biopsy was double-immunostained for TH or PGP9.5 in combination with CD34, a vascular marker of endothelial cells. This to investigate the association of TH+ and PGP9.5+ nerve profiles with vascular structures and bone surfaces. The sections were labeled with polyclonal rabbit anti-TH antibodies (AB158, Millipore) or anti-PGP9.5 antibodies (SAB4503057, Sigma), which were detected with polymeric alkaline phosphatase-conjugated anti-rabbit IgG (BrightVision DPVR-AP, Immunologic), and visualized with Liquid Permanent Red (LPR; Dako K0640). Subsequently, the sections were labeled with mouse anti-CD34 antibody (clone QBEND/10, ab78165, Abcam), which was detected with polymeric horseradish-peroxidase-conjugated anti-mouse IgG (BrightVision DPVM-HRP, Immunologic). The signal was enhanced with DIG-conjugated Tyramide Signal Amplification (TSAPlus DIG, PerkinElmer), which was detected with horseradish-peroxidase-conjugated anti-DIG sheep Fab fragments (11 207 733 910, Roche) and visualized with Deep Space Black (DSB; BRI4015, Biocare Medical).

All sections were counterstained with Mayer’s hematoxylin and mounted with Aquatex (Merck). All primary antibodies were diluted in Renoir Red diluent (PD904, Biocare Medical), as this enhanced the antibodies binding. Skin biopsies were included as positive controls for the PGP9.5 and TH immunostaining. Unspecific staining of secondary antibodies and visualization systems were investigated by the omission of primary antibodies.

### Microscopy and imaging

The immunostained sections were viewed and imaged with an upright DMRXAZ Leica microscope (Leica, Wetzler, Germany) with an Olympus UC30 camera using the Olympus cellSens entry software, version 1.14. The Masson’s trichrome-stained sections were viewed and imaged under polarized light with the same microscopic system to separate the quiescent, eroded and osteoid (formative) surfaces (Fig. 4b). The eroded and formative surfaces were collectively defined as the remodeling surfaces. The extent of these surfaces was marked on printed maps (x2.5 images) of the biopsies, as previously described^69^, to guide the histomorphometric analysis of the nerve densities above quiescent and remodeling surfaces. The markings were validated by a second observer.

### Histomorphometry

The histomorphometric analysis was conducted on scans of the immunostained sections. The scans were made with a NanoZoomer Slide Scanner (Hamamatsu, Japan), and analyzed using NDP.view2 software (Hamamatsu Photonics K.K., EU). A point-grid was mounted on the screen to estimate the size of the tissue areas in which the nerves were investigated. The local nerve densities were estimated as the number of nerves per area (mm^2^) instead of, the more common, nerve fiber length per volume (mm^3^), since it was not possible to cut and immunostain 30–50-µm-thick sections from the available human bone biopsies, and because this estimate was stereologically less affected by the direction of the nerve fiber. None of the two estimates would allow one to fully address the branching and connection of the nerves, as these branches and connections may be present above or below the plane of the histological section.

For the sequential double-immunostaining with TH and PGP9.5, the number of nerve profiles per tissue area (nerve profile density) and the proportion of the nerve profiles associated with vasculature were estimated within the bone marrow, cortical pore and periosteal compartment (Fig. 1a). The analysis was conducted on scans of section 2, allowing the observer to separate the TH+ and the TH−/PGP9.5+ nerve profiles (Fig. 1b–d). Each nerve profile was marked within the scans and given an identification number, facilitating that the identified nerve profiles could be reviewed by a second observer. The point grid was used to estimate the respective areas of the three sub-compartments, with each point representing an area of 0.024 mm^2^. Accordingly, the number of nerve profiles per tissue area was calculated as the sum of nerve profiles divided by the number of grid-points hitting the sub-compartment and the constant of 0.024 mm^2^/point.

For the double-immunostaining of either TH or PGP9.5 in combination with CD34, the number of nerve profile per tissue area in the bone marrow and their association to the given vasculatures and bone surfaces were estimated. Here the analysis was conducted on scans of section 3–4, allowing the observer to investigate the TH+ and PGP9.5+ nerve profiles separately and address their association with the CD34 immunostained vasculature. Each identified nerve profile in the bone marrow was marked with an arrow within the scans, allowing a second observer to validate the nerve profile and its association with a given vasculature.

The vasculatures were separated based on their size and morphology: (i) Large arteries (arterioles) were defined as vessels lined with more than two layers of smooth muscle cells; (ii) small arteries were defined as vessels lined with one or two layers of smooth muscle cells; (iii) capillaries including post-capillary venules were defined as thin vessels (diameter <15 µm) often lined with pericytes and not lined with smooth muscle cells; and iv) sinusoids were defined large diameter vessels with a thin endothelial lining and no lining with smooth muscle cells (Fig. 3a–h). Nerve profiles identified surrounding a vascular structure were grouped together as a cluster profile, i.e. a cluster profile represents an individual vascular structure, which can be innervated by multiple nerve profiles.

The investigated bone marrows were subdivided into different zones: The bone marrow above 100 µm of the bone surface and the bone marrow within 100 µm of the bone surface, which was further divided into zones above quiescent surfaces and above remodeling surfaces (Fig. 4a,b). The number of nerve profiles, cluster profiles and grid-points within these zones was counted, and the number of nerve profiles or cluster profiles per area of bone marrow (#/mm^2^) in the different zones was calculated, as described above.

### Statistical Analysis

Statistical analysis was performed with GraphPad Prism (version 6.01 GraphPad Software Inc.) and StataIC (version 10, StataCorp LP, College Station, TX). For all statistical analyses, a probability value of p < 0.05 was considered significant.

The statistical significance of the difference between the numbers of nerve profiles per tissue area within the three sub-compartments was assessed using repeated measure Analysis of Variance (ANOVA) with Geisser-Greenhouse’s correction to correct for unequal variances. The statistically significant difference between the number of TH+ relative to TH−/PGP9.5+ nerve profiles in the different sub-compartments, as well as the number of nerve profiles associated with vascular structures in the different sub-compartments, was analyzed using clustered logistics regression analysis. Here all the identified 1171 nerve profiles were taken into consideration, where the nerve profiles from the individual subjects were clustered. More precisely, this analysis addressed whether the odds between number of nerve profiles and the size of the defined bone compartments were statistically significant, i.e. having an odds ratio different from zero when taking into consideration that each patient represents an individual cluster and that all patients (clusters) may not have the same number of observations.

The statistical significance of the difference in the number of nerve or cluster profile per marrow area, as well as the number of nerve profiles per cluster profile between the defined zones (above versus within 100 µm of the bone surface, and above quiescent versus remodeling surfaces), were analyzed using clustered logistics regression analysis. More precisely, this analysis addressed whether the odds between the number of nerve or cluster profiles and the number of grid-points within the defined zones of the bone marrow was statistically significant. Similarly, the analysis address whether the odds between numbers of nerve profiles over numbers of cluster profiles is significantly different between the defined zones.

## Data Availability

All data obtained during this study are available upon request.
